# The LILRB family in hematologic malignancies: prognostic associations, mechanistic considerations, and therapeutic implications

**DOI:** 10.1186/s40364-024-00705-7

**Published:** 2024-12-19

**Authors:** Alan Hodges, Rachel Dubuque, Shu-Hsia Chen, Ping-Ying Pan

**Affiliations:** 1 Center for Immunotherapy, Neal Cancer Center, Houston Methodist Research Institute, Houston, TX 77030 USA; 2 Texas A&M University College of Medicine, Bryan, TX 77807 USA; 3 Department of Physiology, Biophysics, and Systems Biology, Weill Cornell Medical Science and Graduate School of Medical Sciences, New York City, NY 10065 USA

**Keywords:** LILRB, ILT, LIR, PIRB, Leukemia, Lymphoma, Myeloma, CAR T-cells, ADCs, ICT

## Abstract

The leukocyte immunoglobulin-like receptor B (LILRB) proteins, characterized by their transmembrane nature and canonical immunoreceptor tyrosine-based inhibitory motifs (ITIM) signaling, play a pivotal role in maintaining immune homeostasis and are implicated in the pathogenesis of various disease states. This comprehensive review will focus on the intricate involvement of the LILRB family in hematologic malignancies. These receptors have emerged as valuable diagnostic and prognostic biomarkers in leukemia, lymphoma, and myeloma. Beyond their prognostic implications, LILRBs actively shape the immune microenvironment and directly influence the disease pathogenesis of hematologic malignancies. Furthermore, their identification as potential therapeutic targets offer a promising avenue for precision medicine strategies in the treatment of these disorders. Currently, multiple LILRB directed therapies are in the preclinical and clinical trial pipelines. This review underscores the multifaceted role of the LILRB family in hematologic malignancies, highlighting their significance from diagnostic and prognostic perspectives to their broader impact on disease pathophysiology and as valuable therapeutic targets.

## Background

Cancer immune evasion is a hallmark of cancer progression. During cancer development, malignant cells interact and silence host-immune cells enabling cancer to circumvent immune recognition and targeting. These intricate networks between cancer cells and their surrounding immune ecosystem are still not completely understood, presenting challenges in the development of effective modern cancer therapies. The success of recent immune checkpoint blockade therapies has energized cancer immunologists and clinicians to investigate and counteract the immune-suppressive pathways that are permissive to cancer growth. Identification and classification of immunoreceptors that modulate the tumor microenvironment is critical in understanding disease progression and driving engineering of new immunotherapies.

**Fig. 1:**
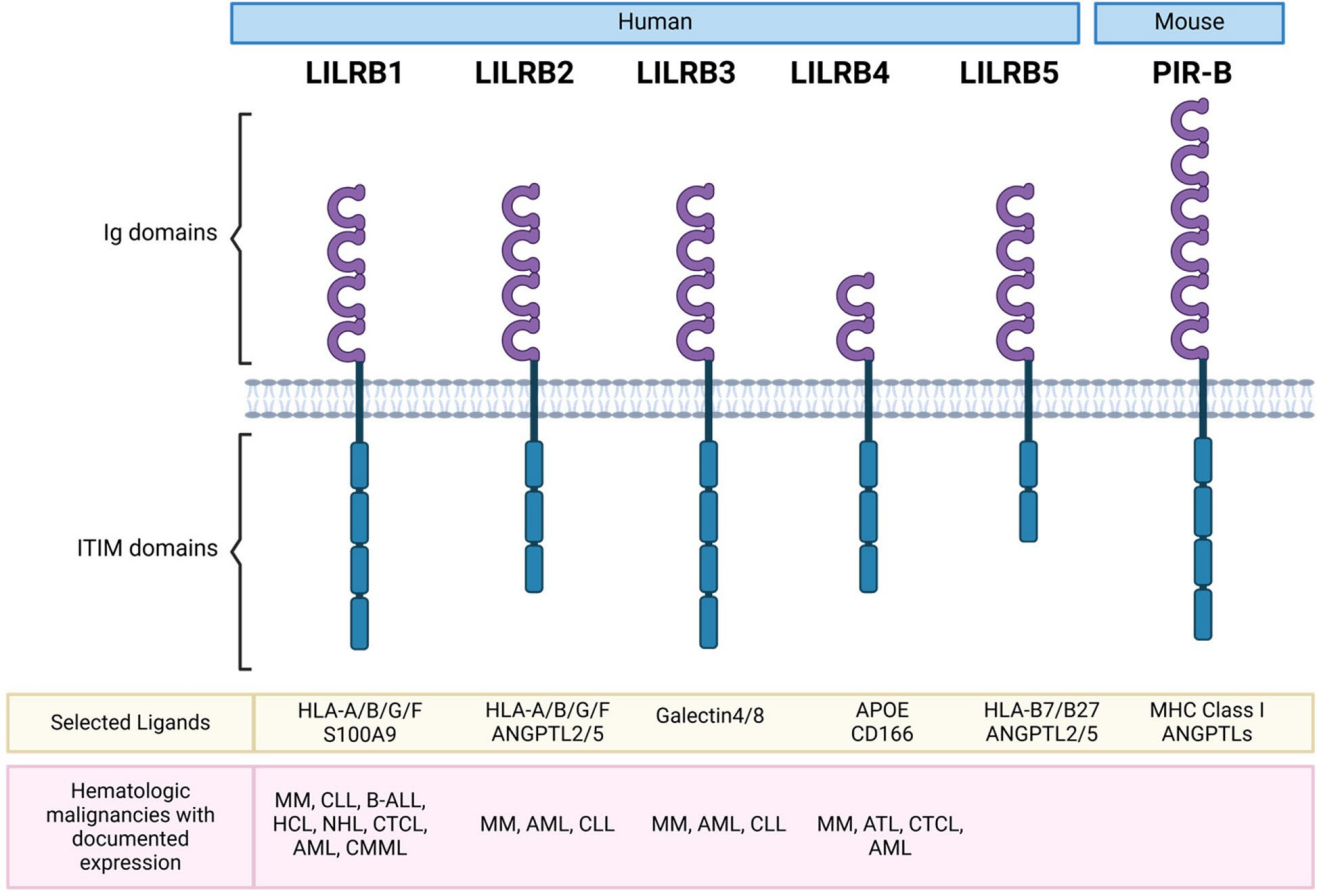
LILRB family structure, selected ligands, and hematologic malignancy profile. Human LILRB family and structure, notable ligands, and malignancies with documented LILRB surface expression. LILRB mouse ortholog PIR-B structure and ligands are also noted. A more exhaustive list of ligands has been reviewed elsewhere [1–7]. Created in BioRender. Dubuque, R. (2025) https://BioRender.com/c05n053

**Fig. 2:**
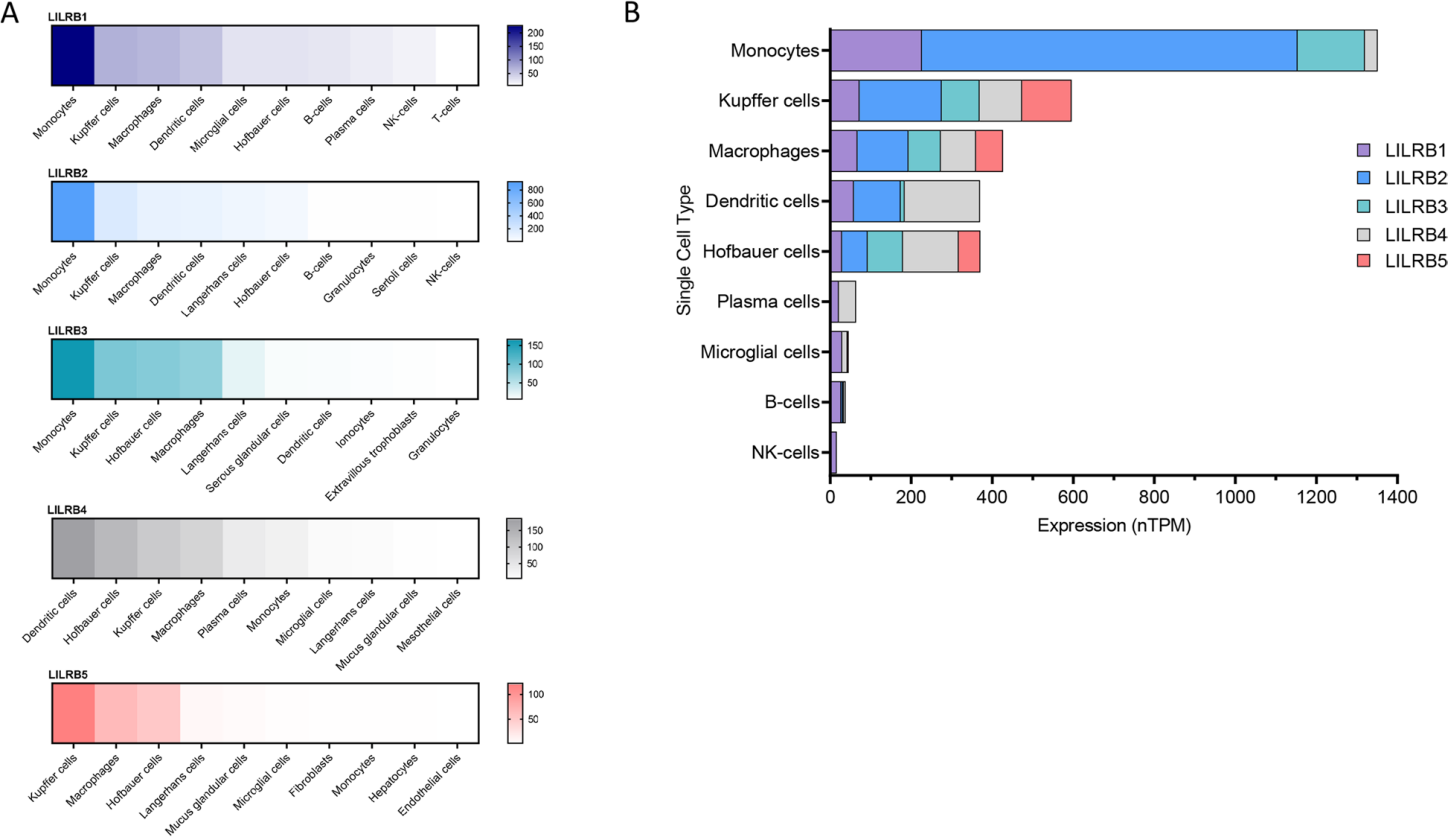
LILRB family expression across tissue types. **A** RNA expression of each individual member of the LILRB family across tissue types. **B** Comparison profile of all LILRB members across tissue types. Single cell expression data compiled from the Human Protein Atlas

A family of inhibitory immunoreceptors, the leukocyte immunoglobulin-like receptor B (LILRB) family, has been implicated in the progression of advanced hematologic malignancies and other diseases states which have been previously reviewed (Fig. 1) [1–7]. LILRBs have been demonstrated to modulate professional antigen-presenting cell (APC) function, suppress cytotoxic immune targeting of cancer cells, and initiate reprogramming of the tumor immune microenvironment. The LILRB family consists of 5 members LILRB1 (LIR-1, ILT2, CD85j), LILRB2 (LIR-2, ILT4, CD85d), LILRB3 (LIR-3, ILT5, CD85a), LILRB4 (LIR-5, ILT3, CD85k), LILRB5 (LIR-8, CD85c) [7]. The LILRB family has variable expression among cell types, however, is most prominently expressed by hematologic cells of myeloid and lymphoid origin, with limited expression in other tissues (Fig. 2) [1, 5, 7]. This family of ITIM containing transmembrane receptors was first described in the 1990s by the work of Pulford et al. [8], Colonna et al. [9], and Cosman et al. [10]. Significant progress has been made in characterizing LILRB cellular expression patterns, identifying receptor ligands, exploring physiological mechanisms, and developing LILRB targeted therapeutics. Currently, multiple LILRB directed therapies are in preclinical and clinical trial pipelines. This review seeks to thoroughly explore and discuss the LILRB family in the context of hematologic malignancies with documented surface LILRB expression, spotlighting their significance from diagnostic and prognostic perspectives to their broader impact on malignant pathology and as valuable therapeutic targets.

## LILRB family expression as diagnostic and prognostic biomarkers in hematologic malignancies

### B-cell malignancies

LILRB1, LILRB2 and LILRB4 have been described as diagnostic markers in B-cell malignancies. LILRB1 has been shown to have a unique “V-shaped” expression pattern across the B-cell development lifecycle [11] with a high-low–high pattern of LILRB1 expression observed of many B-cell maturity markers including CD34, CD20, CD45 and CD10. Investigators have used “Loss of V-shaped pattern” as diagnostic for B-cell acute lymphoblastic leukemia (B-ALL) with all normal samples displaying the normal pattern and all B-ALL samples displaying an aberrant pattern. Although yet to be confirmed in an independent cohort, this is a promising method for B-ALL diagnosis, especially in the determination of measurable residual disease (MRD). Interestingly, the same group of investigators further showed LILRB1 as a biomarker for CD19-B-ALL in post-CD19 directed therapy relapsed disease [12], highlighting the utility of LILRB1 in MRD detection.

LILRB2 and LILRB4 were demonstrated to be specific biomarkers of chronic lymphocytic leukemia (CLL) and show consistent expression patterns between the two receptors. In one cohort of patients, 6 LILRB2 + patients were uniformly LILRB4 + and 5 LILRB2- patients were uniformly LILRB4- [12]. LILRB4 was further validated by another group in CLL as specific marker distinguishing between donor derived healthy donor and CLL samples [13]. Additionally, LILRB4 expression correlated with lymphoid tissue involvement in CLL/small lymphocytic lymphoma (SLL), a prognostic indicator of poor outcomes [14]. High expression of LILRB4 has also been associated with worse outcomes in multiple myeloma (MM) [15].

### Myeloid malignancies

In myeloid malignancies, LILRB1, LILRB3, and LILRB4 have important diagnostic and prognostic roles. LILRB1 and LILRB4 expression, independently, are highly sensitive for monocytic differentiation in acute myeloid leukemia (M-AML) [16–18]. In one case series of 64 cases of M-AML, and 57 cases of AML without monocytic differentiation (NM-AML), LILRB1 and/or LILRB4 positivity distinguished monocytes/monoblasts of M-AML from myeloblasts from NM-AML with 100% sensitivity and specificity [17]. These results are similar to those obtained in earlier cohorts examining LILRB4 only; LILRB4 positivity was observed in all cases of M-AML, and no cases of NM-AML [16, 18]. Furthermore, the use of LILRB4 quantification in terms of florescence intensity and/or RT-PCR may provide additional insights in separating neoplastic myeloid cells from healthy cells. In the cohort of 64 M-AML patients previously described, LILRB4 mean florescence intensity (MFI) in M-AML was significantly higher than on normal monocytes in NM-AML (*p* < 0.001) [17]. Similarly, quantification of LILRB4 expression of bone marrow (BM) stem-cells (CD34 +) samples through RNA sequencing was shown to be greater in chronic myelomonocytic leukemia (CMML) patients compared with healthy controls and myelodysplastic syndrome (MDS) patients, although a diagnostic cutoff was not established [19].

The LILRB family has important prognostic implications in AML. LILRB1-4 expression is associated with increased overall mortality and decreased event free survival in independent AML patient cohorts [20, 21]. Interestingly, a converse trend was observed with LILRB5, which was associated with favorable outcomes in AML patients [20]. LILRB4 expression at diagnosis has also been strongly associated with the development of secondary CNS involvement in AML [22]. Although M-AML has already been demonstrated to have increased risk of central nervous system (CNS) development [23], the marginal utility of LILRB4 positivity to prognostic models beyond FAB/WHO classification may be limited. Notably, LILRB4 expression was not associated with increased mortality in these patients.

Although LILRB expression is generally indicative of poor prognosis in myeloid malignancies, LILRB high expressing disease may be more treatment sensitive. LILRB expression was correlated with expression signatures of predicting immune checkpoint blockade ICB response in the TCGA AML dataset [20]. Moreover, LILRB4 expression has been correlated with a trend in increased responsiveness to hypomethylating agent (HMA) therapy in CMML and MDS, although the sample size was too small to demonstrate statistical significance [19]. Interestingly, HMA therapy has been shown to increase LILRB4 expression in AML multiple cell lines [19, 24]. As both immune checkpoint blockade (ICB) therapy in AML and HMA therapy in myeloid malignancies have shown limited efficacy [25–27], the use of LILRB expression as a guide therapy selection or inform HMA/ICB combinatorial therapy [28] design may enhance therapeutic efficacy.

Future work evaluating LILRB expression in other hematologic malignancies is warranted as current data is largely focused on AML. Additional work to standardize these diagnostic endeavors through comparison of the wide variety of commercial LILRB specific antibody clones would improve the quality and generalizability of each result.

## Role of the LILRB family in the immune microenvironment of hematologic malignancies

The LILRB family has widely documented roles in the immune microenvironment of both solid tumors and hematologic malignancies. In solid tumors, LILRBs have been shown to inhibit the function of cytotoxic NK-cells, T-cells, and contribute to a pro-tumor myeloid compartment [1, 3]. Undoubtedly many of these functions/roles of LILRB proteins demonstrated in solid tumor models play in the immune microenvironment of hematologic malignancies as well.

### T and NK cell populations

**Fig. 3:**
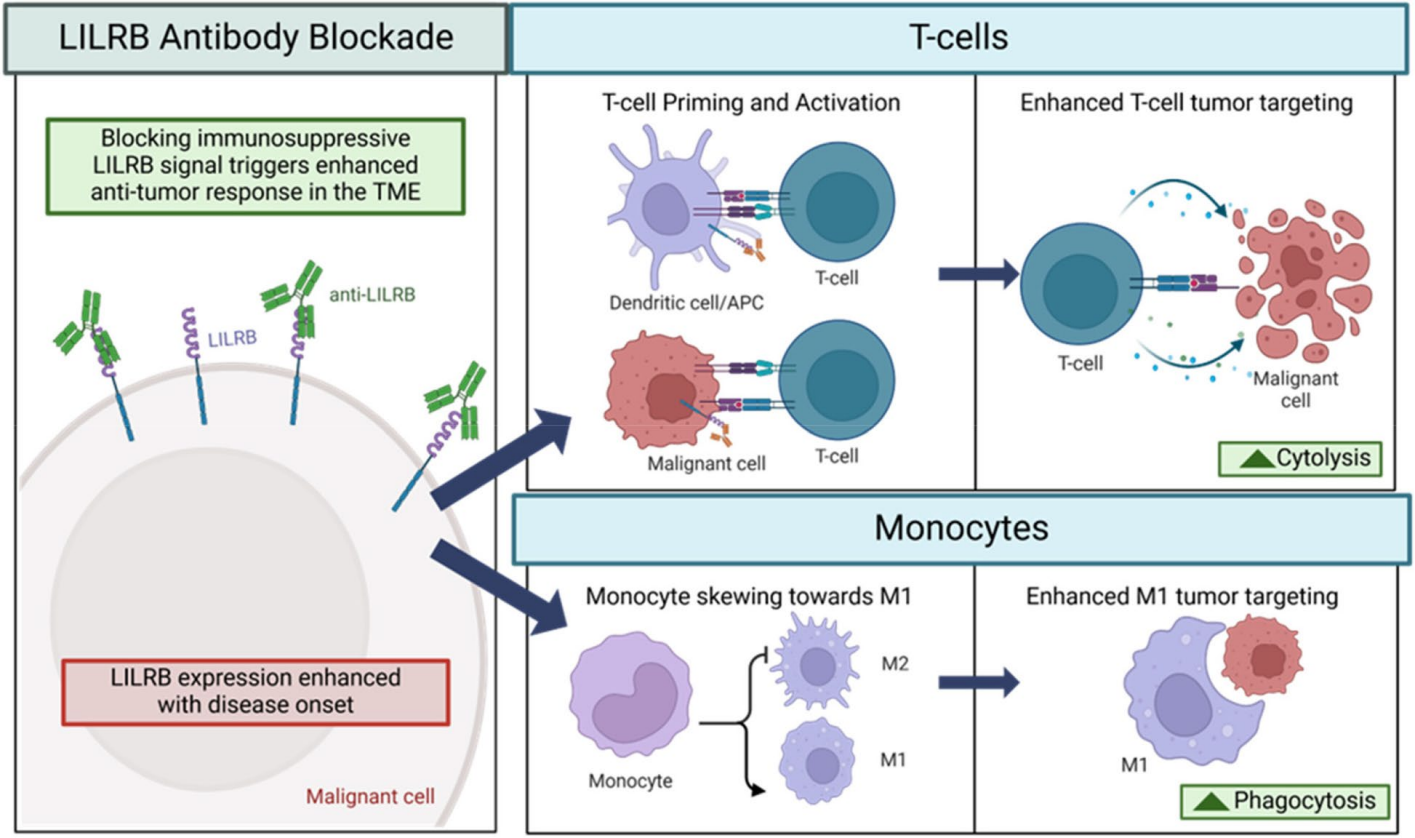
LILRB blockade in the tumor microenvironment**.** LILRB blockade prevents immunosuppressive signaling and improves T-cell cytolysis and monocyte phagocytosis in the TME. Created in BioRender. Dubuque, R. (2025) https://BioRender.com/q51d918

LILRB1 blocking antibodies have been used to explore the biology of LILRB1 mediated immune suppression in hematologic malignancies. LILRB1 blockade increases cytolysis of NK-cells and T-cells (Fig. 3). LILRB1 is expressed on a significantly larger proportion of NK-cells in patients with multiple myeloma and CLL than in healthy donors [29]. Similarly increased expression of LILRB2/LILRB3 was observed in NK-cells of patients with AML [30]. LILRB1 antibody blockage increased killing of primary AML, ALL, and CLL and MM cell lines. This phenomenon was first described when the LILRB1 blocking antibody HP-F1 restored the diminished NKL lysis 721.221 B-lymphoblast cell line following transfection with class I human leukocyte antigens (HLA) [9]. This effect has been recapitulated in primary AML and ALL cells. LILRB1 antibody blockade increased polyclonal allogeneic NK-cell activation, degranulation and cytolysis of primary AML and ALL cells in vitro [31]. Simultaneous dual blockade of NKG2A and LILRB1 further enhanced cytotoxicity. Similarly, LILRB1 mAb blockade enhanced in vitro killing of the K562 cell line as observed from ex vivo NK-cells derived from CLL patients [32]. LILRB1 blockade was shown to improve cytolytic activity of NKL cells against multiple malignant B/T-cell lines in vitro and the MM cell line KMS27 in vivo [29]. Notably however, LILRB1 antibody blockade did not increase the NK-92 cell line mediated cytolysis of MM cell lines [33].

LILRB1 has also been shown to be a major inhibitor of Vγ9Vδ2 T-cells and antibody blockade can restore lytic function of this T-cell subset [34]. Vγ9Vδ2 T-cells have been shown to have potent antitumor activity in hematologic malignancies, although protumor roles have been described as well [35]. LILRB1 blockade increased cytolysis of the Vγ9Vδ2 T-cell clone G42 and the SIL polyclonal Vγ9Vδ2 T-cell population against the MM cell line RPMI 8226 [34]. Despite adoptive transfer of Vγ9Vδ2 T-cells showing preclinical efficacy, clinical trials have had underwhelming efficacy [36, 37]. Modulation of LILRB1 in this setting is a potential avenue to improve Vγ9Vδ2 T therapy. In an unsupervised approach to identify costimulatory molecules which could potentiate Vδ2- γδ T-cell response, LILRB1 blockade was found to dramatically reduce Vδ2- γδ cytolysis of large B-cell lymphoma cells, with no effect on Vδ2 + γδ T-cells [38]. This highlights the importance of careful application of LILRB directed therapy, as unexpected pleiotropic responses in different cell populations such as Vδ2- γδ T cells may occur.

### Myeloid populations

Additionally, LILRB1 blockade has been demonstrated to increase macrophage phagocytosis (Fig. 3). Knock out of LILRB1 on macrophage promoted M1 macrophage differentiation, and when combined with CD47 blockade, increased the phagocytosis of tumor cell lines [39]. Conversely, CD47 blockade in LILRB1 WT cells had minimal effect. Notably, the M1 macrophage skewing was present even without coculture, indicative of cis HLA I-LILRB1 interaction on the macrophage. This finding was translated to increase the antibody dependent cellular cytotoxicity (ADCC) of the anti-CD20 antibody rituximab in primary B-cell malignancy samples through dual antibody blockade of CD47 and LILRB1 [40]. A notable trend of increased expression of LILRB1 BM macrophages has been observed in diffuse large B-cell lymphoma (DLBCL) patients who had BM infiltrating disease compared with those who do not [40]. Similarly, an increased concentration of LILRB4 in cell culture supernatant was observed when myeloma cells were co-cocultured with BM aspirates compared with monocultured BM aspirates [41]. Together these results highlight the potential to reprogram the myeloid rich BM microenvironment through the use of LILRB directed agents.

### Ligand dysregulation

Dysregulation of LILRB ligands also contributes to disease pathogenesis in hematologic malignancies. Non-classic class I HLAs, ligands of LILRB1 and LILRB2, are integral components of the disease microenvironment in multiple hematologic malignancies [42]. One non classical HLA I, HLA-G, is a high affinity ligand of LILRB1 and LILRB2 [43]. Increased levels of HLA-G have been noted in the plasma of patients with various B-cell, T-cell, and myeloid cell malignancies [42, 44]. Interestingly, like LILRB1/LILRB4, serum soluble HLA-G levels are much higher in AML M4/M5 subtypes compared to control serum and serum from AML M1/M2 patients [45]. In CLL, HLA-G expression is positively correlated with immunosuppression and worsened prognosis [46]. Agonism of the LILRB axis is a putative mechanism for immunosuppression observed in these patients.

### The PIRB axis and murine tumor engraftment

Agonizing the LILRB/PIRB axis increased lymphoma implantation in murine models. Treatment of CD34 humanized mice with an LILRB3 agonizing mAb treatment was demonstrated to create a permissible microenvironment for the engraftment of allogeneic patient derived B-lymphoma cells, whereas tumor did not engraft in untreated mice [47]. LILRB3 agonism in this model promoted M2 skewing of primary CD14 + monocytes, a phenotype favoring xeno-engraftement. Similarly, in immune competent Balb/c mice, Fon lymphoma xenograft HLA-G expression, purportedly acting through the LILRB ortholog PIRB, conferred engraftment advantage over HLA-G- lymphoma [48]. Mice with HLA + engrafted tumors, showed marked reduction in peripheral T-cell populations with decreased proliferative capacity and a concurrent increase in peripheral blood MDSC populations.

## Neoplasm intrinsic expression of the LILRB family in the pathogenesis of hematologic malignancies

Direct expression of LILRB family proteins on malignant cells themselves further modulate immune cell-tumor cell interaction. This interplay adds much nuance to the field of tumor immunotherapy in hematologic malignancies as immune stimulating therapies may also stimulate proliferation of malignant subsets as well and vice-versa with immunomodulating agents.

### Myeloid malignancies

Although LILRB expression has been noted on multiple myeloid neoplasms including MDS, AML and chronic myeloid leukemia (CML), the contribution of malignant cell expression of the LILRB family in disease pathogenesis has been most thoroughly examined in AML. Expression of each of the LILRB1-4 members and PIRB on AML cells have been implicated in contributing to the pathogenesis of the disease. LILRB expression on AML cells is associated with a multitude of pathogenic mechanisms including altering proliferation and maturation of AML cells and also contributing inducing an immunoinhibitory environment. Most simply, LILRB expression contributes to AML cell proliferation, as demonstrated with shRNA-mediated LILRB1-4 expression inhibiting proliferation of MV4-11 cell line [49]. Additionally, CLPs-miR-103a-2-5p microRNA was demonstrated to suppress LILRB3 expression levels inhibiting AML growth [50]. LILRB3 blocking antibodies were also shown to inhibit proliferation of LILRB3 transduced U937 and HL-60 cells [21].

LILRB signaling has been shown to induce stem-like properties in AML. As the seven-member angiopoietin-like proteins (ANGPTL) family had been previously shown to promote the hemopoietic repopulation ability of HSCs acting through LILRB2 and PIRB [51, 52], the effects of this signaling axis were examined in AML. In the premature myeloid cells of AML, PIRB contributes to in the MLL AF9 murine leukemia model. PIRB WT MLL-AF9 demonstrated an increased stem like transcriptomic profile with decreased myeloid differentiation compared to PIRB deficient MLL-AF9 [51]. PIRB WT MLL-AF9 engrafted mice also demonstrated markedly reduced survival compared to PIRB deficient MLL-AF9 engrafted mice, demonstrating the impact to pathogenesis of increased AML stemness. LILRB3 has likewise been associated with reduced maturation in AML cells [21].

The AML cell intrinsic consequences of LILRB3 have recently been well explored through the use of antagonist and agonist antibodies to the receptor as well as LILRB3 KD/KO. LILRB3 has been shown to promote AML progression, survival, immune evasion, and maturation blockade [21, 53]. LILRB3 antibody agonism inhibited AML cell death in in vitro, while in vivo AML LILRB3 expression led to increased AML engraftment and mortality in immunodeficient and immunocompetent AML mouse models [53]. Mechanistically, LILRB3 was shown to act as an activating receptor, inducing NF-κB signaling through recruitment of TRAF2 and cFLIP, in addition to the canonical inhibitory activity mediated through ITIM-SHP1/SHP2 [53]. Separately LILRB3 antagonism was demonstrated to promote a more mature myeloid phenotype acting through induction of CEBP genes, IRF genes, JUNB, and PU.1. LILRB3 antagonism also inhibited phosphorylation of AKT and mTOR signaling, important mediators of AML pathogenesis [54]. Notably as Akt can be a downstream target of NF-κB [55], a unified signaling regime incorporating both described mechanisms is likely. As identifying the natural ligand of LILRB3 has been elusive, antibody ligation of LILRB3 has been used to explore the biology of this receptor. Recently galectin4/7 has been identified as a potential ligand and therefore more biologically relevant models can be utilized [56].

In addition to induction of an aggressive AML disease state, LILRB signaling in AML contributes to an immunosuppressed and AML permissive microenvironment. LILRB3 signaling in AML cells inhibits T-cell activity, with LILRB3 agonism of AML cells inhibiting subsequent cytolysis by cocultured T-cells [56]. LILRB3 antibody agonism of AML cells promoted the expression of immunoinhibitory markers including IL10 and CD163 [21].The pro-survival benefit of anti-LILRB3 antibodies observed in humanized mice was of CD8 T-cells abolished the pro-survival effects of anti-LILRB3 antibodies significantly but not completely abrogated with CD8 depletion, highlighting the crucial contribution immune inhibition plays in LILRB3 mediated pathogenesis [53].

Similar inhibition of T-cell responses has been observed in AML [57, 58]. LILRB4 + AML cells suppressed T-cell proliferation compared with LILRB4- AML cells from the same patient [57]. In T-cell coculture experiments, LILRB4 KO in AML cell lines increased in vitro cytotoxicity, which was restored with reintroduction of full length LILRB4 [57]. Further work examining the role of each ITIM in LILRB4 revealed that only 2/3 ITIMs (Y412/Y442) were essential T-cell inhibition whereas Y360 was not [58]. Notably, in the same system LILRB1 KO was not associated with decreased T-cell proliferation.

LILRB4 expression increases transendothelial migration of AML cell lines [57]. NSG mice transplanted with LILRB4 KO THP-1 cells demonstrated increased infiltration of solid organs including liver, spleen, and BM. Increased migration in vitro, and increased organ involvement in vivo due to LILRB4 expression were recapitulated with syngeneic C1498 cells in immunocompetent mice. The authors implicate the LILRB4–SHP-2–NFκB–uPAR–ARG1 axis in mediating increased migration and T-cell suppression in AML. ARG1 is an important mediator in myeloid cell mediated immune inhibition in AML [59, 60], in this model the malignant myeloid cells of AML can further contribute to MDSC-like immune-inhibition. Interestingly however, unlike inhibition of T-cell activity, all three ITIMs of LILRB4 were found to contribute to increased leukemia cell infiltration [58]. One potential upstream regulator of LILRB4 expression is protein arginine methyltransferases 5 (PRMT5). Knockdown of PRMT5 has been shown to decrease AML cellular adhesion and migration, acting at least partially through the downregulation of LILRB4 [61]. Interestingly, knockdown of PRMT5, and therefore inhibition of LILRB4 signaling, decreased the phosphorylation of AKT and mTOR, pathways similarly affected by LILRB3 antagonism.

Corresponding to this mechanistic data, AML patient sample transcriptomics support a role of LILRB in the production of an immunoinhibitory microenvironment [20, 62]. LILRB1-4 expression is correlated with other markers of immune-inhibition including CD300, Tim-3 VISTA and CD86 [62]. Furthermore, LILRB1-4 expression was inversely correlated with infiltrating immune cells [63]. Thus, LILRB expression in AML promotes a protumor microenvironment in AML.

In the myeloproliferative neoplasm (MPN) polycythemia vera (PV), it has been shown that HLA-G inhibits the formation of PV patient derived erythropoietin-independent erythroid colonies and decreased the proliferation of UT7/EPO and HEL cell lines [64]. The authors did not detect either LILRB1 or LILRB2 on the surface of UT7/EPO or HEL cell lines, leading them to conclude HLA-G acts on another uncharacterized receptor.

### Lymphoid malignancies

Expression of one or more LILRB member has been noted on a variety of lymphoid neoplasms including both B-cell, T-cell, and NK-cell malignancies [8, 33, 65, 66]. Notably, the CD85 molecule, was originally defined Fifth Workshop on Leucocyte Antigens in 1993 by VMP55 and GHI/75 antibody binding discovered through immunization of mice with glycoprotein enriched lysate of hairy cell leukemia [8, 67]. Later these antibodies were discovered to be specific for ILT2 (LILRB1). Since, the role of LILRBs malignancies has been greatly expanded, however LILRB1 remains the most widely elucidated in this context.

In B-cell malignancies, LILRB1 may play an important role in controlling the proliferation and progression of neoplasm. The LILRB1 ligand HLA-G inhibits the proliferation of B-cell lymphoma, MM, and pre-B-cell leukemia cell lines. Neoplastic B-cell growth is impaired by HLA-G/ILT2 interaction [68]. In the Raji B-cell lymphoma cell line, HLA-G inhibited proliferation through induction of G0/G1 cell-cycle arrest and inhibition of the PKC and AKT/mTOR pathways. Importantly this HLA-G mediated proliferation inhibition was partially abrogated through LILRB1 siRNA knockdown and antibody blockade, implicating LILRB1 as an important negative regulator of neoplastic B-cell growth. Similarly, LILRB4 has been shown to inhibit BCR-induced AKT activation, inhibiting the progression of CLL [13].

Lozano et al. strongly implicate LILRB1 in multiple pathogenic mechanisms in MM [69]. The authors observed that LILRB1 and the LILRB1 ligand s100a9 mRNA expression was decreased in MM, and the MM precursor MGUS, compared to plasma cells derived from healthy donors. Furthermore, in CD138 + plasma cells, a decrease in both mRNA and surface protein expression of LILRB1, LILRB2 and LILRB3 was decreased in myeloma vs MGUS. In addition to these observational data, overexpression of LILRB1 in myeloma cell lines induced downregulation 13 out of 116 genes whose products are involved in the pathogenesis and progression of MM. Furthermore, forced LILRB1 overexpression in MM increased cord blood derived NK-cell (cb-NK) and T-cell mediated cytotoxicity. Together, these results implicate loss of LILRB1 expression as a mediator of disease progression, upregulating MM associated genes, and reducing the susceptibility of MM cells to immune cytolysis.

T-cell malignancies have been documented to express HLA-G and LILRB1, including in primary cutaneous CD8 + and CD56 + T-cell lymphomas and peripheral circulating Sézary cells [65, 70]. In a cell line derived from a cutaneous T-cell lymphoma (CTCL), LILRB1 was found to constitutively associate with SHP-1 phosphatase [70], a negative regulator of TCR signaling [71]. TCR signaling components are mutated in 84% of Sezary syndrome samples and TCR signaling is widely considered oncogenic in these cells [72]. Interestingly, LILRB1 expressing cells from 1 Sezary Syndrome patient were less susceptible to CD3/TCR-dependent activation-induced cell death [52], an anti-oncogenic role of TCR also noted in adult T-cell lymphoma patients [72]. Thus, LILRB1 may inhibit the proliferation of CTCL but induce resistance to activation-induced cell death.

Interestingly, soluble and/or surface bound LILRB4 may also play as a ligand, with inverse implications in T-cell lymphoma and healthy T-cell mediated immune response. Soluble LILRB4 has been noted in the supernatant of MM-BM cocultured cells [41], as well as multiple solid tumor malignancies [73]. The use of exogenously delivered soluble LILRB4-Fc fusion protein has been shown to inhibit T-cell leukemia growth and decrease leukemic burden in vivo [63]. LILRB4-Fc fusion protein was shown to bind CD166/activated leukocyte cell adhesion molecule (ALCAM) present on activated T-cells and inhibit malignant cell proliferation through the p70S6K signaling pathway [63]. Somewhat similarly, LILRB4-Fc fusion protein has been shown to inhibit T-cell responses and decrease the T-cell immune response through induced anergy [74]. A similar T-cell suppression effect of LILRB4 was observed in LILRB4 expressing MM cells, where KO of LILRB4 on MM cell lines promoted T-cell proliferation [15].

Lymphoid neoplasms display the contradictory actions that can be induced through LILRB modulation. As malignant cells of immune origin share many similar pathways as their nonmalignant counterparts, agents promoting inhibition of tumor growth, may also inhibit anti-tumor immune response. In addition to the case of soluble LILRB4 in T-cell malignancies/T-cell mediated immunity, the HLA-G/LILRB1 axis in B-cell malignancies well illustrates this phenomenon. Whereas HLA-G has been shown to impair the growth of neoplastic B-cells acting through LILRB1 [68], LILRB1 blockade increases NK-cell cytolysis and macrophage phagocytosis of B-cell malignancies [9, 29–31, 39]. Therefore, careful applications of LILRB directed therapies are warranted, after consideration of both potential pro and anti-tumor effects, in order to find the most suitable patient subsets for these therapies.

## Direct tumor targeting of LILRB family proteins in hematologic malignancies

Many characteristics of the LILRB family make them favorable targets for antibody and cellular therapies. The tissue restricted pattern of LILRBs lends limited on-target, off-tumor side effects of LILRB targeted agents. The inducible nature of LILRBs in hematologic malignancies allows for the design of many combinatorial therapeutic strategies. LILRB1 is preferentially induced by lenalidomide in leukemic B-cells compared with healthy B-cell controls [32]. LILRB3 has been shown to be induced by IFNy, polyIC, and cytarabine in AML cell lines and patient samples [21], while hypomethylating agents and fat mass and obesity-associated protein (FTO) induced LILRB4 expression in AML cells [19, 24]. LILRBs represent attractive therapeutic targets for both single and multi-agent strategies with many currently in the developmental pipeline.

Monoclonal antibody and effector T-cell therapies are currently in preclinical and/or clinical trials. Here we will highlight multiple therapeutic agents currently in development which directly target tumor LILRB expression. Of note multiple clinical trials examining the use of LILRB blocking antibodies as immune checkpoint therapies are ongoing (LILRB1: NCT04913337, NCT04717375; LILRB2: NCT03564691, NCT04669899, NCT05054348, NCT03564691; LILRB1/2:NCT04913337), however none are enrolling patients with hematologic malignancies. LILRB directed therapies for hematologic malignancies are listed in Tables 1 and 2.

**Table 1:** LILRB specific monoclonal antibodies in clinical trial for use in hematologic malignancies

Therapeutic Agent	Target	Disease	Trial Phase	Trial number
*IO-202*	LILRB4	r/r AML, r/r CMML	Phase 1, recruiting	NCT04372433
*MK-0482*	LILRB4	r/r myelomonocytic or monoblastic/monocytic AML, r/r CMML	Phase 1b, terminated	NCT05038800

**Table 2:** LILRB specific effector T-cells in clinical trial for use in hematologic malignancies

Therapeutic Agent	Target	Disease	Trial Phase	Trial number
anti-ILT3 CAR-T	LILRB4	r/r AML(M4/​M5)	Phase I, recruiting	NCT04803929
anti-ILT3 STAR-T	LILRB4	r/r AML, (LILRB4 + BM)	Phase I, completed	NCT05518357
anti-ILT3 STAR-T	LILRB4	r/r AML (LILRB4 + BM)	Phase I, not yet recruiting	NCT05548088
anti-ILT3 STAR-T	LILRB4	r/r AML, r/r CMML (LILRB4 + BM)	Phase I, not yet recruiting	NCT05739409

### LILRB specific monoclonal antibody-based therapies

Multiple anti-LILRB4 antibody-based therapies are in the development pipeline for AML. Antibody blockade was originally noted to inhibit T-cell suppression and increase AML migration secondary to AML LILRB4 expression described above [57]. Anti-LILRB4 mAb treated mice had decreased tumor burden and increased survival compared with IgG, demonstrated in both immunocompetent and humanized PDX mouse models [75]. Humanized LILRB4 antibody h128-3 has been shown promote LILRB4 internalization and subsequent degradation through multiple mechanisms which contribute to increased functional antagonism of the receptor, however may also contribute to target antigen loss in the AML cell [76]. Notably, two peptide inhibitors of LILRB4 have also been produced to mimic the LILRB4-h128-3 interaction [77]; the smaller size and/or PK parameters of peptide inhibitors may afford for unique future LILRB4 directed therapies.

Currently two anti-LILRB4 mAb are in clinical trial for AML, IO-202 a humanized IgG1 monoclonal antibody and MK-0482 a humanized IgG4 mAb (Table 1). Preliminary results from NCT04372433 enrolling 46 patients utilizing IO-202 as monotherapy and in combination with azacytidine, report IO-202 demonstrated encouraging safety and efficacy results including 1 ongoing CR in an LILRB4 high expressing AML patient undergoing combination therapy [78].

In addition to traditional monoclonal antibodies, an antibody drug conjugate (ADC) [79] and a bispecific T-cell engager (BiTE) [80] targeting LILRB4 have been developed. LILRB4 monoclonal antibodies derived from h128-3 with monomethyl auristatin F (MMAF) payloads were produced with varying drug-antibody ratio (DAR). Such ADCs demonstrated increased in vitro AML cell line killing as well as increased survival in a THP1 xenograft mouse model. Another non-traditional antibody therapeutic NGM936, a BiTE bispecific for CD3 x LILRB4, has been reported for the treatment of AML in a published abstract [80]. Authors report NGM936 induced T-cell mediated cytotoxicity of AML cell lines and patient derived AML cells and decreased circulating leukemic burden in vivo in a humanized AML PDX mouse model. NGM936 has also demonstrated preclinical efficacy in MM and demonstrated induced activation of healthy donor T-cells cocultured with post-BCMA CAR T relapsed patient myeloma cells.

As with other immune checkpoint monoclonal antibody therapies [81], LILRB4 blocking antibodies treatment have been associated with at least one case of an immune related adverse event (IRAE) in an AML patient. A 48-year-old woman with therapy related MDS and subsequent AML transformation 180 days post-allogeneic hematopoietic cell transplantation developed myocarditis twelve-days following one dose of 75 mg MK-0482 [82]. Similarly, myositis was observed in 2 patients in a receiving MK-0482 in combination with pembrolizumab in a solid tumor clinical trial cohort [83]. The preliminary report for IO-202 for r/r AML/CMML reported treatment-related adverse events including gastrointestinal symptoms, headache, chills, and infusion related reactions [78]. All of these adverse events were from the monotherapy group, and none were reported in the IO-202-azacytidine combination group. These data highlight the need for continued evaluation of the safety profile of anti-LILRB4 monoclonal antibodies.

Currently clinical trials of LILRB directed therapies are limited to LILRB4 (Table 1). However, observational and preclinical data suggest LILRB3 may have therapeutic potential in AML [3, 21, 53, 66]. In a cohort of 108 patients receiving HSCT, 5.4% of patients developed LILRB3 reactive antibodies, which are not found in healthy patients [66]. These anti-LILRB3 antibodies were demonstrated to arise from mismatches in the polymorphisms of LILRB3 between HSCT donor and recipient. These results demonstrate that anti-LILRB3 antibodies may be an important mediator of GVL effects, and therefore a potential target for exogenously administered antibodies. In addition to induction of immune mediated effects, multiple groups have demonstrated preclinical use of LILRB3 blocking antibodies to inhibit AML proliferation and progression [21, 53]. LILRB3 blockade decreased the AML disease burden of LILRB3 expressing MLL-AF9 leukemia in immunocompetent mice and an AML PDX in humanized mice [53]. Notably, LILRB3 blockade has been shown to reduce the T-cell inhibition of MDSCs in solid tumor models [56]. LILRB3 monoclonal antibody-based therapeutics are of interest preclinically and warrant further investigation.

### LILRB specific effector T-cell based therapies

The first demonstration of LILRB CAR T was described by John et al. in 2018 [84]. Consistent with other results LILRB4 was shown to be highly expressed on FAB M5 subtype, with limited expression in normal tissues or HSCs. LILRB4 specific CAR T-cells demonstrated in vitro cytotoxicity against AML and LILRB4 monocytes. In vivo efficacy has been exemplified in MV4-11 AML xenograft and KMT2Ar mutated ALL/AML lineage switch models, showing decreased leukemic burden and prolonged survival [85]. Subsequently, the development of LILRB4 specific nanobody-based Synthetic T-Cell Receptor and Antigen Receptor-T (STAR-T) cells have demonstrated cytotoxic activity against LILRB4 + AML cell lines in vitro and corresponding anti-leukemic activity in vivo [86]. Clinical trials of LILRB4 specific CAR T-cells and STAR T-cells are ongoing (Table 2).

Like monoclonal antibodies, only LILRB4 directed effector T-cells are currently under clinical trial, however LILRB3 directed CAR T-cells are in preclinical development for AML [21]. LILRB3 CAR T-cells decreased leukemic burden and increased survival in an LILRB3 + MV411 xenograft model, and decreased BM leukemic burden in an autologous CAR T PDX model. Moreover, although yet to be demonstrated, LILRB2 has been identified as potential CAR T target and LILRB2 was expressed by approximately 76% of cells in most AML patient specimens. LILRB2 complementary target LILRB2 + CLEC12 stained on average 93% of AML cells while staining < 5% of normal HSCs and T-cells [87].

## Conclusions and future directions

The LILRB family have many important prognostic, mechanistic, and therapeutic implications in hematologic malignancies. Although much has been reported in the past 25 years, much work remains to be completed in order to characterize these receptors contribution more completely. One critical gap in mechanistic understanding remains examining exactly which contexts the inhibitive signaling of the LILRB family controls neoplastic proliferation, and where conversely the LILRB family contributes to pathogenesis. Additionally, LILRB5 remains woefully unexplored. Currently, high quality commercially available anti-LILRB5 antibodies are not available, a hurdle which must be overcome to increase knowledge of this receptor. Although knowledge gaps remain, therapeutically LILRB family members are highly promising with preclinical development of therapeutic monoclonal antibodies and effector T-cells. Unfortunately, LILRB4 is the only target currently undergoing clinical trial in hematologic malignancies. Further examination of the other LILRB members and the development of clinical products targeting them remains a vital topic for further work.

## Data Availability

No datasets were generated or analysed during the current study.
